# Oxidative DNA Damage and Carotid Intima Media Thickness as Predictors of Cardiovascular Disease in Prediabetic Subjects

**DOI:** 10.3390/jcdd5010015

**Published:** 2018-03-07

**Authors:** Roshan Kumar Mahat, Neelima Singh, Akshara Gupta, Vedika Rathore

**Affiliations:** 1 Department of Biochemistry, Gajra Raja Medical College, Gwalior 474009, India; neelima48@rediffmail.com (N.S.); ved_sin26@rediffmail.com (V.R.); 2 Department of Radiodiagnosis, Gajra Raja Medical College, Gwalior 474009, India; aksharagupta007@gmail.com

**Keywords:** prediabetes, 8-hydroxy-2-deoxy-guanosine, carotid intima media thickness, cardiovascular disease

## Abstract

Prediabetes is considered as a risk factor for the development of diabetes mellitus and cardiovascular disease. The present study was conducted with the aim of finding out the relationship between oxidative DNA damage and carotid intima media thickness for the prediction of cardiovascular disease in prediabetic subjects. The study included 100 prediabetic subjects and 100 normal individuals as controls. In both cases and controls, 8-OHdG was measured by ELISA, and CIMT was measured by B mode ultrasonography. Both 8-OHdG and CIMT were significantly higher in subjects with prediabetes as compared to controls (185.80 ± 10.72 pg/mL vs. 126.13 ± 16.01 pg/mL, *p* < 0.001 and 0.70 ± 0.04 mm vs. 0.57 ± 0.03 mm, *p* < 0.001, respectively). There was significant and positive correlation of IGT with 8-OHdG (r = 0.783; *p* < 0.001) and CIMT (r = 0.787; *p* < 0.001) in prediabetic subjects. Moreover, 8-OHdG showed significant positive correlation with CIMT (r = 0.704; *p* < 0.001) in prediabetic subjects. In conclusion, increased 8-OHdG and CIMT in prediabetic subjects indicate that biochemical changes of atherosclerosis start even before the onset of diabetes mellitus. Hence, 8-OHdG and CIMT could be used as indicators of cardiovascular disease risk in these subjects.

## 1. Introduction

Prediabetes is a condition in which the person suffers from impaired glucose metabolism with an elevated blood glucose level that is not yet high enough to be diagnosed as diabetes mellitus [1]. It is associated with the simultaneous presence of insulin resistance and β-cell dysfunction abnormalities that begin even before the glucose changes are detectable [2]. Prediabetes is considered as a risk factor for developing future diabetes, cardiovascular disease [3], and subsequent adverse cardiovascular events including myocardial infarction [4], stroke [5], and even death [6]. The prevalence of prediabetes is increasing globally. In the year 2010, the prevalence of prediabetes was estimated to be 343 million and is expected to increase 471 million by the year 2035 [7].

The American Diabetes Association (ADA) recognizes the following value ranges for identifying individuals with prediabetes: fasting plasma glucose level from 100 mg/dL to 125 mg/dL or 2 h plasma glucose value after 75 gm oral glucose during oral glucose tolerance test (OGTT) 140 mg/dL to 199 mg/dL. A glycated hemoglobin (HbA1c) level ranging from 5.7% to 6.4% is further considered as diagnostic criteria of pre-diabetic condition [8].

There is considerable evidence that hyperglycemia results in the production of reactive oxygen species (ROS), which ultimately ends up in the induction of oxidative stress [9,10]. Oxidative stress is caused by excess production of ROS and free radicals beyond the physiological quenching capacity of the cells or their ability to repair the resulting damage. At low to moderate concentration, ROS and free radicals play an important role in biological systems, such as cell signaling, controlling vascular tone, and the generation and degradation of target cells, but their high concentration causes damage to the macromolecules lipids, proteins, and DNA [11]. Of the molecules that are subjected to oxidative modification, DNA has received the greatest attention. A plethora of products are formed in DNA from the reaction of reactive oxygen species with purines and pyrimidines. Among purine and pyrimidine base, guanine is more vulnerable to oxidation. The hydroxyl radical can assail to the C-8 position of guanine and generate an oxidation product, 8-hydroxy-2-deoxy-guanosine (8-OHdG) [12]. 8-hydroxy-2-deoxy-guanosine is considered to be the most sensitive and specific biomarker of oxidative stress-induced DNA damage, and repair and is also an early marker of endothelial dysfunction [13,14].

Atherosclerosis is a generalized phenomenon and is more or less present equally within the coronary, cerebral, and carotid arteries. Therefore, ultrasonographic evaluation of easily accessible arteries has been considered as a surrogate marker for less accessible arteries, such as coronary and cerebral arteries [15]. Ultrasound imaging provides information regarding intima media thickness, presence, and type of plaque, calcification, and wall diameter. This information enabled assessment of pre-symptomatic lesions, induration of the arteries burden, and reduced death and disabilities from CVD [15,16]. Subclinical atherosclerosis develops several years prior to the clinical manifestation of cardiovascular disease. If the patient can be diagnosed with vascular injury at the subclinical stage, the preventive measure can be implemented to limit future vascular complications [17,18]. Carotid intima media thickness (CIMT) is a well-known, non-invasive, inexpensive, rapid, and reproducible measure, and increased CIMT has been considered as a recognized surrogate marker for assessing cardiovascular risk and recognizing subclinical atherosclerosis [17,19].

To the best of our knowledge, the role of oxidative DNA damage and carotid intima media thickness for prediction of cardiovascular disease in prediabetes has not yet been documented in the literature. Hence, the present study was designed to address this lacuna by measuring 8-hydroxy-2-deoxy-guanosine (as a marker of oxidative DNA damage) and carotid intima media thickness (as an indicator of subclinical atherosclerosis) in prediabetic subjects.

## 2. Materials and Methods

### 2.1. Study Design and Subjects

This was a cross-sectional study and was carried out in the Department of Biochemistry, Gajra Raja Medical College, Gwalior, India after obtaining ethical clearance from the Institutional Human Ethics Committee. 100 subjects with prediabetes of either sex (age group 20–55 years) and 100 normal healthy individuals of same age group were enrolled in the present study. They were selected from general population, and those who were at risk of developing diabetes (who had at least one of the main risk factors for diabetes—first degree relative with diabetes, BMI ≥ 25 kg/m^2^, women who were diagnosed with gestational diabetes mellitus, women with polycystic ovary syndrome, persons who are physically inactive, and other clinical conditions associated with insulin resistance e.g., severe obesity, acanthosis nigricans, etc.) in Gwalior City were given a predesigned screening questionnaire.

The diagnosis of prediabetes was made on the basis of the following criteria:

(a) Fasting plasma glucose level 100–125 mg/dL (IFG) and
(b) 2-h plasma glucose (after giving 75 gm of glucose) level 140 mg/dL to 199 mg/dL (IGT).

Those with a normal range of blood glucose level had been selected as the control group (FBS < 100 mg/dL, 2-h plasma glucose concentration after giving 75 gm of glucose <140 mg/dL).

Patients of type 2 diabetes mellitus, hepatic disease, cardiovascular disease, renal disease, pulmonary tuberculosis, acute or chronic inflammatory illness, gout and arthritis, prolonged illness, subjects not willing to give consent or refuse to participate in the study, and patients receiving medicines known to alter glucose metabolism were excluded from the present study.

All subjects had given their written informed consent for participation after a full explanation of the study.

### 2.2. Anthropometric Measurements

Body weight was measured using calibrated electronic weighing scales in the morning prior to eating, and height was measured to the nearest centimeter by employing a portable stadiometer without shoes. Body mass index (BMI) of the subjects was calculated using standard formula, BMI = Weight (Kg)/[Height (m)]^2^. Waist circumference (WC) was measured utilizing an anthropometric tape at a level on the skin midway between the mean point of iliac peak and the inferior border of the last rib at the level of the umbilicus while in a standing position at the end of gentle expiration. Hip circumference (HC) was measured over the widest part of the gluteal region at the level of pubic tubercle in standing position. Waist to Hip ratio (WHR) was calculated by waist circumference (cm) divided by hip circumference (cm).

### 2.3. Blood Pressure Measurements

Systolic blood pressure (SBP) and diastolic blood pressure (DBP) were taken using a standardized mercury sphygmomanometer using standard recommended procedures.

### 2.4. Biochemical Measurements

Fasting blood sample was collected after at least 10–12 h of overnight fasting for biochemical investigations. After that, 75 gm of glucose was given orally to each participant, and plasma glucose concentrations were measured at 120 min during OGTT. Plasma glucose was estimated using the glucose oxidase and peroxidase (GOD-POD) method by using a commercially available kit from ERBA Diagnostics Mannheim, Germany on Mindray BS-400 chemistry analyzer. HbA1c in whole EDTA blood was estimated using the turbidimetric immunoassay method by using a commercially available kit from APTEC Diagnostics. 8-OHdG in serum was estimated by ELISA using a commercially available kit from Cloud-Clone Corp, (Katy, TX 77494, USA).

### 2.5. Measurement of CIMT

The CIMT was determined by using a high-resolution B mode ultrasonography system with an electrical linear transducer mid-frequency of 7.5 MHz. The patients were examined in the supine position with the neck extended and the probe in the anterolateral position. The intima media thickness was measured as the distance between first and second echogenic lines. The first echogenic line delineates the lumen intimal interface, and the second echogenic line was produced by the collagen containing an upper layer of the tunica adventitia. All measurements were performed by the well-trained sonographer who was blinded to all clinical data of the patients.

### 2.6. Statistical Analysis

Data were analyzed using Statistical Package for Social Science version 20 (IBM, SPSS Statistics 20, Armonk, NY, USA), and graphs were generated with the help of GraphPad Prism 5 and Microsoft Excel. The data were checked by Shapiro-Wilk test for normal distribution. The statistical differences between cases and controls were determined by student independent sample *t*-test and Mann-Whitney U test for normally distributed data and skewed data, respectively. Categorical variable was analysed with the help of chi-square test. Pearson’s correlation was used to verify the relationship between studied parameters. The results were considered significant if *p* < 0.05.

## 3. Results

**Figure 1:**
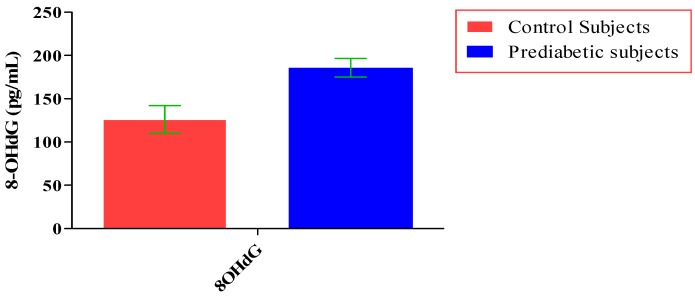
Showing comparison of 8-OHdG between prediabetic and control subjects (data presented as mean ± SD).

**Figure 2:**
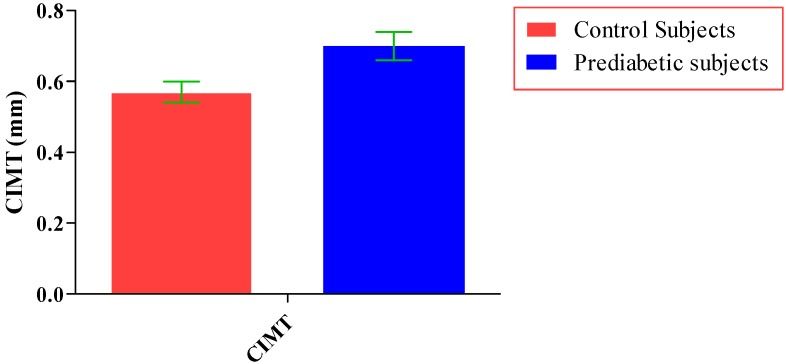
Showing changes in CIMT due to impaired glucose tolerance in prediabetic subjects (data presented as mean ± SD).

**Figure 3:**
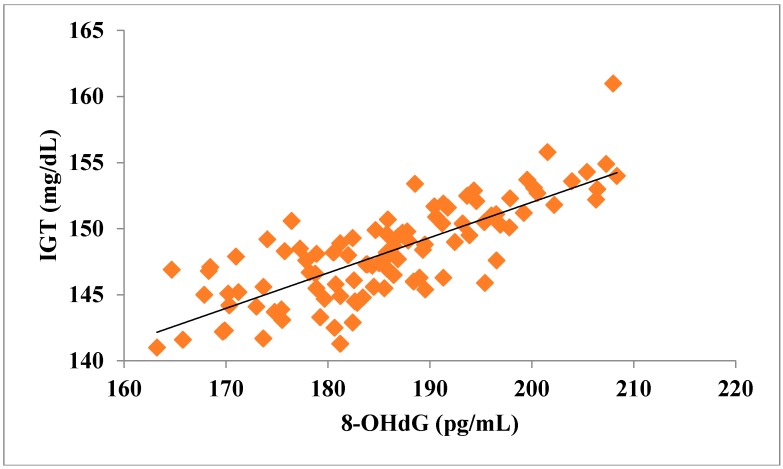
Showing correlation of IGT with 8-OHdG in prediabetic subjects (r = 0.783; *p* < 0.001).

**Figure 4:**
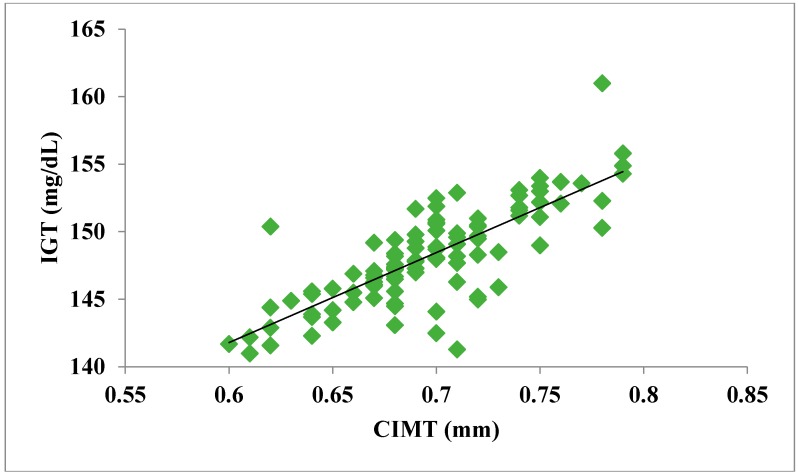
Showing correlation of IGT with CIMT in prediabetic subjects (r = 0.787; *p* < 0.001).

**Figure 5:**
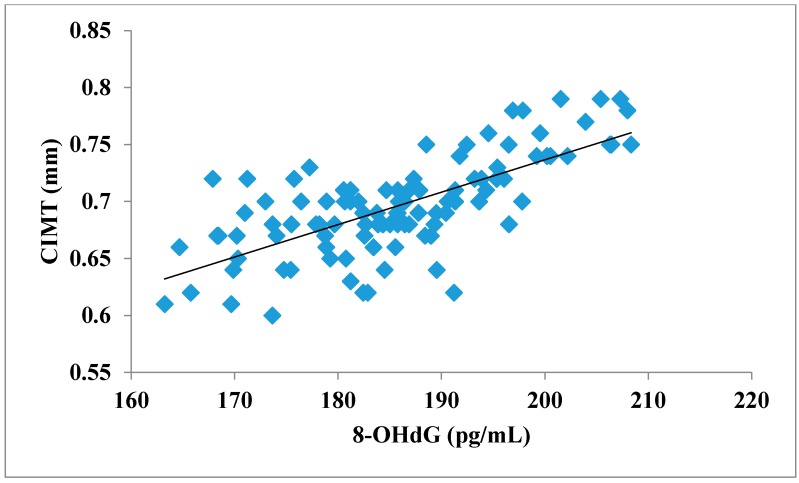
Showing correlation between 8-OHdG and CIMT in prediabetic subjects (r = 0.704; *p* < 0.001).

The total number of subjects included in the present study was 200, out of which 100 were prediabetic subjects and 100 were age-matched controls having normal blood glucose. Table 1 shows the demographic data of prediabetic patients and controls. The mean age of prediabetic and control subjects was 39.43 ± 8.90 years and 38.92 ± 7.85 years, respectively, with no significant difference, indicating that the subjects with both the groups were age-matched. There was no significant difference in sex distribution between prediabetic and control subjects. Prediabetic patients had significantly higher mean BMI, WC, HC, and WHR than the control subjects, indicating that prediabetic subjects had higher rate of general obesity (based on BMI) and central obesity (based on WHR). Table 2 shows biochemical parameters and carotid intima media thickness of studied subjects. Both CIMT and 8-OHdG were significantly higher in prediabetic subjects as compared to control subjects (0.70 ± 0.04 mm vs. 0.57 ± 0.03 mm, *p* < 0.001 and 185.80 ± 10.72 pg/mL vs. 126.13 ± 16.01 pg/mL, *p* < 0.001, respectively). Table 3 shows Pearson’s correlation analysis between IGT, 8-OHdG, and CIMT in prediabetic subjects. 8-OHdG was significantly and positively correlated with IGT (r = 0.783; *p* < 0.001) in prediabetic subjects. Similarly, CIMT was significantly and positively correlated with IGT (r = 0.787; *p* < 0.001). In addition, there was also a significant positive correlation between 8-OHdG and CIMT (r = 0.704; *p* < 0.001) in prediabetic subjects. Table 4 shows Pearson’s correlation analysis of 8-OHdG and CIMT with age, BMI, and WHR in prediabetic subjects. 8-OHdG was significantly and positively correlated with age (r = 0.486; *p* < 0.001), BMI (r = 0.450; *p* < 0.001), and WHR (r = 0.368; *p* < 0.001) in prediabetic subjects. Similarly, CIMT was also significantly and positively correlated with age (r = 0.469; *p* < 0.001), BMI (r = 0.344; *p* < 0.001) and WHR (r = 0.241; *p* < 0.05) in prediabetic subjects. Figure 1 shows comparison of 8-OHdG between prediabetic and control subjects. Figure 2 shows changes in CIMT due to impaired glucose tolerance in prediabetic subjects. Figure 3 and Figure 4 show correlation of impaired glucose tolerance with 8-OHdG and CIMT, respectively, in prediabetic subjects. Figure 5 shows correlation between 8-OHdG and CIMT in prediabetic subjects.

## 4. Discussion

Prediabetes is the intermediate state of abnormal glucose regulation that lies between normal blood glucose levels and type 2 diabetes mellitus and has been considered as a risk factor for diabetes mellitus and cardiovascular disease (CVD) [20]. In this cross-sectional study, we assessed the relationship between CIMT (as an indicator of subclinical atherosclerosis) and 8-OHdG (a marker of oxidative stress-induced DNA damage) in subjects with prediabetes in order to know the risk of cardiovascular disease in these patients.

In our study, we found increased levels of glucose (both fasting and 2 h plasma glucose after giving 75 gm of glucose) in prediabetic subjects as compared to control subjects. In addition to this, we also found an increased level of HbA1c in prediabetic subjects as compared to controls. Consistent with our findings, Mohieldein et al., 2014 reported a significant increase in the levels of glucose and HbA1c in prediabetic subjects as compared to normoglycemic subjects [21]. Furthermore, in our study, besides having hyperglycemia, prediabetic subjects have significantly higher levels of BMI and WHR, which are considered as indicators of general obesity and central obesity, respectively. These results are in agreement with the findings of Agarwal et al. [22] and Ferrannini et al. [23], who reported that prediabetic subjects have mild hyperglycemia, increased BMI, WC, and increased WHR compared to normal control subjects, which predispose prediabetic subjects to an increased risk for cardiovascular disease (CVD).

Hyperglycemia results in auto-oxidation of glucose, which contributes to the non-enzymatic glycation of proteins and stimulation of polyol pathway. These metabolic changes may lead to an increase in ROS generation, which causes damage to the DNA, including oxidized bases and DNA strand breaks with an increase in 8-OHdG and destruction of endothelial function resulting in atherosclerosis [24,25]. Our study showed a statistically significant increase in the level of 8-OHdG in prediabetic subjects as compared to control subjects, which is in line with previous studies [14,25,26]. The result of the present study indicates cellular damage occurs even before the onset of full-blown diabetes, which is the stage of prediabetes. This may be due to an increased level of glucose in prediabetic subjects, which causes increased formation of ROS that leads to oxidative DNA damage [25]. The increased level of HbA1c in prediabetes may also have a purported impact on DNA damage. Hydrogen peroxide, which is generated during hyperglycemic condition, induces the release of iron from hemoglobin and induces more release from glycated hemoglobin than from its non-glycated analog. Free iron participates in Fenton reaction, producing ROS, creating the environment of oxidative stress, and acting as one of the key DNA damaging agents [24,27]. DNA damage induces apoptosis or programmed cell death and endothelial cell death in an early event of atherogenesis that triggers formation of plaque [28]. Also, apoptosis of vascular smooth muscles cells (VSMCs) is associated with the growth of plaques as a result of outward remodelling [29]. Moreover, it triggers intense intimal inflammation, which induces foam cell formation via the accumulation of lymphocytes [30]. Formation of atherogenic lesions may be initiated in arterial smooth muscle cells by mutational events after DNA damage and plaque might progress through an initiation-promotion process [31]. Therefore, 8-OHdG is associated with atherosclerosis and plaque formation [11]. The increased level of 8-OHdG in the prediabetic subjects strongly indicates that not only oxidative damage is occurring but possible microvascular and/or macrovascular disease is already present sub-clinically [32,33]. Pearson’s correlation analysis revealed a statistically significant positive correlation between impaired glucose tolerance and 8-OHdG in prediabetic subjects. This finding of our study supports the fact that hyperglycemia is associated with an increased production of free radicals in the mitochondria and may contribute to a greater DNA damage [10].

Carotid artery intima media thickness can be considered as an excellent non-invasive measure of generalized atherosclerosis and also a surrogate marker of coronary artery disease [34,35,36]. According to INTERNATIONAL ATHEROSCLEROSIS PROJECT, carotid and cerebral arteries and the aorta undergo the atherosclerotic process approximately at the same age as the coronary arteries [37]. Moreover, there is a good agreement between histological examination and the ultrasonographic analysis of coronary arteries [38]. We found significant increased CIMT in prediabetic subjects as compared to controls. This is in accordance with the findings of Altin et al. [17], Bhinder et al. [39], Karbek et al. [40], and Aydin et al. [41]. A thickened CIMT does not immediately lead to cardiovascular events but reflects the degree of atherosclerosis elsewhere in the arterial system [42]. The result of the present study clearly indicates that atherosclerotic process begins well before the clinical onset of full-blown diabetes in individuals with prediabetes.

A correlation analysis showed that CIMT was significantly and positively correlated with IGT in prediabetic subjects. Similar to our finding, Altin et al. [17] and Aydin et al. [41] also found a significant positive correlation of CIMT with blood sugar in prediabetic subjects. This may be due to the creation of oxidative and nitrosative stress in hyperglycemic condition, which acts on the arterial wall to initiate the thickening process [43]. Gomez-Marcos et al. showed a positive correlation of CIMT with fasting plasma glucose, postprandial glucose, and HbA1c and concluded that the patients who present with a metabolic glucose alteration have more of a risk of developing carotid target organ damage [44]. We found that both 8-OHdG and CIMT were significantly and positively correlated with BMI and WHR in prediabetic subjects. These results indicate that obesity is an important risk factor for cardiovascular disease. Apart from this, we also found significant and positive correlation of 8-OHdG and CIMT with age, indicating that as the age advances, the risk for CVD increases.

To the best of our knowledge, this was the first study that has investigated both 8-OHdG and CIMT together among prediabetic subjects and found out the relationship between them. The result of the present study clearly demonstrated that the CIMT as a marker of subclinical atherosclerosis was significantly positively correlated with DNA damage in prediabetic subjects. Hence, in the present study, we demonstrated that 8-OHdG may be a good biomarker for the assessment of the risk of subclinical atherosclerosis in prediabetic subjects.

## 5. Conclusions

In conclusion, the results of our study showed that the biochemical changes of atherosclerosis start even before the onset of diabetes mellitus, i.e., in the prediabetic state, which was indicated by increased 8-OHdG and CIMT. These biochemical changes, along with increased BMI and WHR, may predispose prediabetic subjects to increased risk of cardiovascular disease. Furthermore, our study demonstrates that increased CIMT among prediabetic subjects was associated with increased 8-OHdG. Hence, 8-OHdG could be used to detect atherosclerosis at a subclinical stage in order to mitigate the cardiovascular risk in prediabetic subjects. However, there is a need for further clinical studies in order to explain the role of oxidative DNA damage and CIMT as predictors of cardiovascular disease in prediabetes.

## Figures and Tables

**Table 1:** Demographic data of control and prediabetic subjects.

Variables	Control Subjects (*n* = 100)	Prediabetic Subjects (*n* = 100)
Age (years)	38.92 ± 7.85	39.43 ± 8.90 ^NS^
Sex (M/F)	55/45	51/49 ^NS^
BMI (kg/m^2^)	23.48 ± 1.05	29.07 ± 1.71 **
WC (cm)	82.5 ± 2.84	92.02 ± 3.95 **
HC (cm)	94.85 ± 2.35	95.96 ± 2.66 *
WHR	0.87 ± 0.03	0.96 ± 0.03 **
SBP (mmHg)	116.34 ± 2.94	127.50 ± 5.65 **
DBP (mmHg)	76.50 ± 2.71	81.36 ± 3.60 **

Results are shown as mean ± SD; BMI: Body mass index; WC: Waist circumference; HC: Hip circumference; WHR: Waist to hip ratio; SBP: Systolic blood pressure; DBP: Diastolic blood pressure; ^NS^ Non significant; * Significant at *p* < 0.01; ** Significant at *p* < 0.001.

**Table 2:** Biochemical parameters and carotid intima media thickness of studied subjects.

Variables		Control Subjects (*n* = 100)	Prediabetic Subjects (*n* = 100)
Blood glucose (mg/dL)	Fasting	90.13 ± 3.84	115.88 ± 3.48 **
	2-h PG	124.72 ± 6.23	148.20 ± 3.66 **
HbA1c (%)		4.81 ± 0.14	5.81 ± 0.12 **
8-OHdG (pg/mL)		126.13 ± 16.01	185.80 ± 10.72 **
CIMT (mm)		0.57 ± 0.03	0.70 ± 0.04 **

Results are shown as mean ± SD; HbA1c: Glycated hemoglobin; 8-OHdG: 8-hydroxy-2-deoxy guanosine; 2-h PG: 2 h post glucose after giving 75 gm of glucose during OGTT. ** Significant at *p* < 0.001.

**Table 3:** Showing correlation analysis between IGT, 8-OHdG, and CIMT in prediabetic subjects.

Parameters	IGT	8-OHdG	CIMT
IGT	1	0.783 **	0.787 **
8-OHdG	0.783 **	1	0.704 **
CIMT	0.787 **	0.704 **	1

** Significant at *p* < 0.001; IGT: Impaired glucose tolerance; 8-OHdG: 8-hydroxy-2-deoxy guanosine; CIMT: carotid intima media thickness.

**Table 4:** Showing correlation analysis of 8-OHdG and CIMT with age, and BMI and WHR in prediabetic subjects.

Variables	8-OHdG	CIMT
Age	0.486 **	0.469 **
BMI	0.450 **	0.344 **
WHR	0.368 **	0.241 *

* Significant at *p* < 0.05, ** Significant at *p* < 0.001; IGT: Impaired glucose tolerance; 8-OHdG: 8-hydroxy-2-deoxy guanosine; CIMT: carotid intima media thickness; BMI: Body mass index; WHR: Waist to hip ratio.
